# Estimating Visibility of Annotations for View Management in Spatial Augmented Reality Based on Machine-Learning Techniques

**DOI:** 10.3390/s19040939

**Published:** 2019-02-22

**Authors:** Keita Ichihashi, Kaori Fujinami

**Affiliations:** Department of Computer and Information Sciences, Tokyo University of Agriculture and Technology, Tokyo 184-8588, Japan

**Keywords:** mediated reality, modified perception, augmented reality, spatial augmented reality, view management, annotation, projector, machine-learning, feature selection, depth sensing

## Abstract

Augmented Reality (AR) is a class of “mediated reality” that artificially modifies the human perception by superimposing virtual objects on the real world, which is expected to supplement reality. In visual-based augmentation, text and graphics, i.e., label, are often associated with a physical object or a place to describe it. View management in AR is to maintain the visibility of the associated information and plays an important role on communicating the information. Various view management techniques have been investigated so far; however, most of them have been designed for two dimensional see-through displays, and few have been investigated for projector-based AR called spatial AR. In this article, we propose a view management method for spatial AR, VisLP, that places labels and linkage lines based on the estimation of the visibility. Since the information is directly projected on objects, the nature of optics such as reflection and refraction constrains the visibility in addition to the spatial relationship between the information, the objects, and the user. VisLP employs machine-learning techniques to estimate the visibility that reflects human’s subjective mental workload in reading information and objective measures of reading correctness in various projection conditions. Four classes are defined for a label, while the visibility of a linkage line has three classes. After 88 and 28 classification features for label and linkage line visibility estimators are designed, respectively, subsets of features with 15 and 14 features are chosen to improve the processing speed of feature calculation up to 170%, with slight degradation of classification performance. An online experiment with new users and objects showed that 76.0% of the system’s judgments were matched with the users’ evaluations, while 73% of the linkage line visibility estimations were matched.

## 1. Introduction

Augmented Reality (AR) technology enhances the physical world with digital information to supplement reality, which is basically realized by visual information such as texts and graphics [1]. Head-mount displays (HMD) and hand-held displays such as smart phones and tablets are popular devices as see-through AR displays. By contrast, AR technology primarily realized by a video projector is often called “Spatial AR” [2], which is drawing attention due to the improvement of the performance of a projector such as display resolution, color reproduction, and brightness and its unique characteristics. A projector can be used to superimpose virtual objects on or nearby target physical objects directly. This allows spatial information such as pointing a particular object or place to be presented with intended size, as well as visual characteristics of physical world can be changed by projected light. Furthermore, the projected information can be shared with others in a relatively large display space. These characteristics allows applications to be proposed in various domains, which include domestic work [3,4], assembly tasks [5,6,7], education [8,9,10,11], physical training [12], appearance control for visually impaired [13], entertainment [14], etc.

In AR, a textual or graphical label is often used to annotate physical objects including persons and places. Users obtain information by recognizing a label, in which a linkage line or a leader line is used to associate the label with a target object. The layout of a label has an impact on the effect of information presentation and has been studied for a long time in AR domain known as “view management” [15,16,17,18,19,20,21,22], and even in cartography [23]. In the see-through AR, labels are presented in a computer screen by superimposing a video-captured image; however, in spatial AR, labels are projected on the real world directly. Projected light is mixed with the object’s color and can be interrupted by tall objects depending on the alignment of the projector, objects, and viewpoint. Therefore, unique issues are posed, which are not raised in the see-through AR: deformation of projected information and occlusion of projected information from the user [21,24]. In such a case, users can only see a part of the information or may not notice the presence of information at all. So, the user may misinterpret the information, or it may take longer time to reach correct interpretation, which is critical in safety critical systems that requires quick and correct comprehension such as chemistry experiment support systems [9]. In [21], a view management system for spatial AR called Nonoverlapped Gradient Descent (NGD) was presented, which addressed the occlusion problem by calculating non-occluded area based on pre-registered circumscribed cuboid of tabletop objects and extending the linkage line until the non-occluded area was found. The method allowed user’s faster and more accurate interpretation of information compared with traditional view management method for see-through AR display [15]. However, in a dense area where the number of tabletop objects is large, the length of linkage line tends to be long, which not only makes the time to identify a target object long, but also the processing time. Furthermore, in the worst-case scenario, no projection area is found. Therefore, the condition “nonoverlapped” should be relaxed.

In view management, a weighted linear combination of factors that affect visibility is often utilized [15,20]; however, the methods of finding optimum weights have not been shown or dependent on the application designers. In addition, existing approaches try to find suitable positions for labels and linkage lines, in which “suitability” is not defined from the user’s perspective, rather defined indirectly; for example, sentence “an area with small number of edges should be suitable” is indirect definition of suitability because it is not clear how the user feels the situation and what is expected in the user’s behavior. To address these issues, we apply a supervised machine-learning techniques to model the visibility of an annotation using prospective user’s subjective and objective measures. We define the machine-learning task as a classification problem of visibility. In other words, we propose a software sensor to measure the “projectability” of annotations while reflecting legibility from the users’ perspective. The contributions of the article are as follows:

- Supervised machine learning-based view management method for spatial AR is proposed and implemented.
- A user friendly visibility is defined that reflects human’s subjective mental workload in reading projected information as well as objective measures in corectness of reading.
- Visibility classification features are proposed that represent reflective characteristics of the projection surface, the three dimensional properties of physical objects on the projection surface, and the spatial relationship between the objects, the projector-camera systems, and the user’s viewpoint.
- Feature subset is identified that improves processing speed up to 170% with slight degradation of classification performance.
- An online experiment with new users and objects showed that 76.0% of the system’s judgments were matched with the users’ evaluations, while 73% of the linkage line visibility estimations were matched.

The reminder of the article is organized as follows. Section 2 examines related work in terms of view management techniques. In Section 3 describes an overview of the proposed method including definition of visibility. Specification of visibility class estimation features are presented in Section 4 in detail, and data collection for building visibility class estimator is described in Section 5. The visibility class estimator is evaluated in Section 6. Finally, Section 7 concludes the article.

## 2. Related Work

### 2.1. Mediation of Reality

Mann coined a term “mediated reality” as “a general framework for artificial modification of human perception by way of devices for augmenting, deliberately diminishing, and more generally, for otherwise altering sensory input” [25]. AR is a class of mediated reality which aims at superimposing virtual objects on the real world and supplementing reality with artificial elements [1]. By contrast, Virtual Reality (VR) aims at replacing real world with artificial elements completely, and users are immersed into the artificial world. Our work deals with a view management problem in a projector-based AR, in which virtual objects are directly superimposed on physical objects in a form of text and/or images. In this article, we particularly present a method of estimating the visibility of information for proper label placement.

Diminished Reality (DR) is also categorized in mediated reality, which degrades visual functions for a particular purpose (diminish), covers occluding objects with the image captured prior to being occluded (see-through), filling the occluded region with synthesized image patches (inpaint), and overlaps a real object with a virtual object to replace the real object with the virtual one (replace) [26]. In the projection-based AR, annotation placement (projection) in a blind area is an inherent issue [21]. The “see-through” technique seems applicable in this problem, in which the projected and occluded annotation can be seen through the occluded objects by capturing the hidden area and projecting on the occluded object from the second projector on the other side of the first (main) projector. However, this requires precise capture and projection of the hidden area image from the user’s viewpoint, as well as photometrically correct projection. Also, the occluding object itself may have special meaning in a particular situation and thus should not be artificially invisible. Thus, we consider that the see-through technique is not suitable for view management problem.

### 2.2. View Management Method

A number of view management methods have been proposed to improve the visibility of annotations in AR and Virtual Reality (VR) environments. Highly visible information allows faster and more accurate understanding of associated information. View management (VM) is regarded as label layout optimization problem, in which two approaches exist: geometric-based layout and image-based layout.

#### 2.2.1. Geometric-Based Layout

The geometric-based layout was originally utilized in cartography, in which multiple labels for points, e.g., buildings and the top of mountain, lines, e.g., rivers and roads, and areas, e.g., seas and countries, are placed so that they should not overlap with each other and with relevant map elements [23]. In cartography, the coordinates of map elements is given as well as that of labels and linkage lines. So, the degree of overlap can be calculated using the coordinates information, which is to be minimized against various label positions.

In VR/AR environments, the geometric-based approach is utilized if the geometric information of a target object is given, in which the degree of overlap is encoded as *cost funcation* or *penalty function*. The work by Azuma and Furmanski [15] is one of the earliest work in view management for AR in which they consider overlap of label with other (virtual) objects, labels, and linkage lines, as well as overlap of a linkage line with other linkage lines. They assign different costs for the types of overlap in the cost function. Bell et al. proposed view management techniques for VR environments, where occlusion of labels with virtual 3D objects were resolved to relocate such overlapped labels to visible area [16]. Makita et al. represented the degradation of visibility by the area of overlapped labels and the length of overlapped linkage lines, and the length of linkage line itself [20]. Shibata et al. assigned priority to objects and labels, which is used to resolve the overlap; a label with lower priority is relocated when overlap is detected [27].

Iwai et al. proposed a label placement technique for a projection-based AR in nonplanar and textured surfaces [11], where they capture the projection surface into a computer using RGB and depth-cameras and simulate the legibility of text at various viewpoints around the surface to find the best position for people at different viewpoints. At simulation, the occlusion of projected text with the projection surface, geometric deformation caused by the projection onto nonplanar surfaces, and the contrast lowered by the texture of the reflective properties of a projection surface were estimated. The method shares the goal of ours; however, it demands computational resources pretty much for real-time geometry modelling and label placement calculation. Sato and Fujinami proposed a view management method for spatial AR [21], in which the blind area and occlusion caused by tall physical objects were considered by calculating non-occluded area based on pre-registered circumscribed cuboid of tabletop objects and extending the linkage line until non-occluded area found. As described above, the geometric-based label layout method assumes that the objects’ 2D/3D models are known, which limits the applicability in real world AR systems. VisLP method employs the image-based label layout mentioned below.

#### 2.2.2. Image-Based Layout

The other label-layout approach is image-base layout, in which the “suitability” of rendering information is determined based on the analysis of the background image on which the label is super-imposed. Various factors have been proposed to assess the suitability not only in the context of AR, but also in an issue of general user interfaces such as desktop computer screens (monitors), see-through displays, and video projectors. Scharff et al. showed that the text contrast and the spatial frequency content of the background textures affects the readability of text [17]. The importance of background textures is also identified by Gabbard et al. in the context of outdoor use of see-through display [18], where not only the background textures, but also the changing outdoor illuminance values and text drawing styles, e.g., the text color, the background color, had impact on text identification performance. Relative darkness and uniformity of an area was used by Orlosky to estimate viable regions for text rendering in see-through display environments, which was calculated against gray-scale images [19]. A color-based viewability estimation was proposed by Tanaka, et al. [22], where averages of RGB components, S component in HSV color space, and Y component in YCbCr color space, were used. Leykin and Tuceryan used the contrast between the text and the background and the background texture information derived by Gabor filters. In addition, they used the font size and the font weight [28]. Visual saliency map [29] is often used to highlight prominent regions in an image that attracts human [30,31]. In addition to saliency map, edge information is used to identify unsuitable region for label rendering in [32,33].

In projection-based display systems, Siriborvornratanakul and Sugimoto proposed a method to determine an appropriate vertical area for projection that avoids cluttered area [34]. To identify cluttered areas, Laplacian filters are used, which means that the exact shape information of objects are not used. Riemann et al. investigated a projective interface FreeTop to find suitable tabletop area for projecting information [35]. FreeTop generates “projectability map” based on RGB and depth images, as well as user-defined masks, where information about color edge and lightness are obtained from RGB images, and information of height difference in physical objects are obtained by depth edge from a depth-image. Cotting and Gross proposed an environment-aware display bubble, in which a suitable projection area is identified based on the analysis of reflection properties and its depth discontinuities [36]. They used gray-scale cameras to capture the projection surface appearance of structured imperceptible lights from a projector and applied Gabor filter to highlight continuous surfaces with optimal reflection properties. Similar to [21], these works aim at avoiding overlap of projected information. Although they can avoid overlapping information with objects, it is impossible to avoid being hidden from the user’s eyes because the user’s viewpoint is not considered for estimating the suitability like [11,21].

Similar to the cost function in the geometric-based label layout, the suitability should be measured based on the factors given to the system, and the position that gives the highest suitability measure should be used for a label or an image, and a linkage line if any. The cases with a single factor [17,30,31,34,36] used the value as an indicator of suitability. By contrast, when more than two factors, a linear combination of the factors with appropriate weight is often used, in which the weights are determined empirically or in an top-down manner [19,32], the judgment of the weights depends on the system designers [33,35], or determined in a supervised-manner [11]. A rule-based approach was also proposed by Tanaka et al. [22], where three if-then rules are provided, and the most readable region is determined from three candidates. The linear combination and the rules allows easy interpretation by the system designers; however, the validity of the weights or the rules is a critical issue.

By contrast, Leykin and Tuceryan proposed a text readability estimation method based on machine-learning [28], in which a binary classifier was designed that judges if a particular region in an image with a particular text is readable or not. To train a classifier, six human participants provide ground-truths by experiencing a number of combinations of the background images and the presented texts. In this article, we take the same approach of supervised-machine learning with an extension of projection-based information presentation. Also, our method supports multiple visibility classes, rather than binary classes.

## 3. Overview of Visibility-Aware Label Placement System

### 3.1. VisLP Algorithm

In this section, an algorithm of VisLP is presented to clarify the task of interest. In placing a label, the label is placed so that it should not overlap with other label, which is in common with the NGD method [21]. The fundamental difference between NGD and VisLP is the definition of “visibility”. In the NGD method, it is binary, which means that overlap of a projected label with physical objects is not allowed. By contrast, in case of the VisLP, such overlap is permitted if the “value” of the message is not significantly degraded.

**Figure 1:**
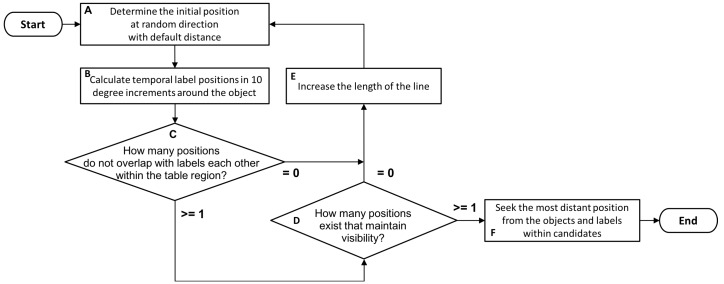
Processing flow of the VisLP algorithm.

Figure 1 shows a processing flow of the VisLP method, which is actually the same as NGD method except for the evaluation of visibility in “D”. When a new object is detected in the field of projection that overlaps with existing labels or a new label placement is requested by an underlying application, the label placement process starts. The initial position of the label is set to be at a location in a random direction and at the default minimum distance (A), and the candidate label positions are set around the target object at increments of 10 degrees (B). After 36 trials, the number of candidate positions without any overlap with labels each other (C) is determined. If there is no candidate without overlap, the length of the linkage line is increased (E). Otherwise, the visibility of candidate position is estimated against the candidates without any overlap, and the number of positions with acceptable visibility is counted (D). If there is no acceptable position, the linkage line is extended (E). By contrast, the position that is the most distant from other objects and labels is chosen as the final answer, i.e., the position of label placement. Note that the acceptability of visibility is judged by the combination of the visibility of both a label and a linkage line. The combination rule depends on the distance between the target object and the label. The condition of acceptance is more relaxed as the distance gets longer, which is to avoid failing in finding acceptable positions in the projection area.

### 3.2. Problem Definition

**Figure 2:**
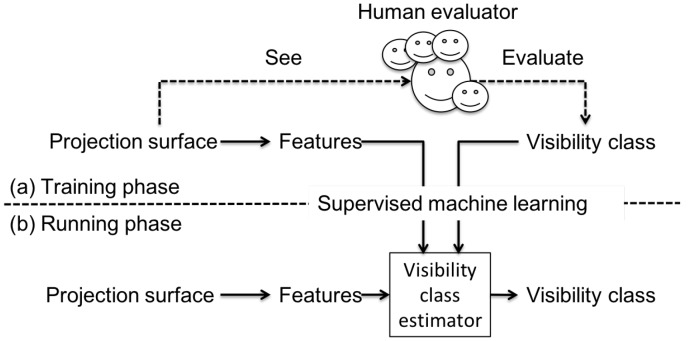
The notion of (a) building (training) an estimator and (b) running online.

The key component of VisLP is estimating visibility based on a supervised machine learning technique. Figure 2 illustrates the notion of building the estimator and using it online. In the training phase (a), we take a collective intelligence approach, where people see a wide variety of label placement situations and evaluate the visibility. The relationship between the situations and the human’s evaluation is learned by a supervised machine learning technique, in which relevant features are actually extracted to represent particular situations, and the evaluation results are discrete classes. In the running phase (b), the built visibility class estimator is used in Figure 1D. In this article, we mainly describe building visibility class estimator, which includes the definition of visibility classes and the design of features for estimation with their offline and online evaluation.

### 3.3. Factors that Degrade Visibility

**Figure 3:**
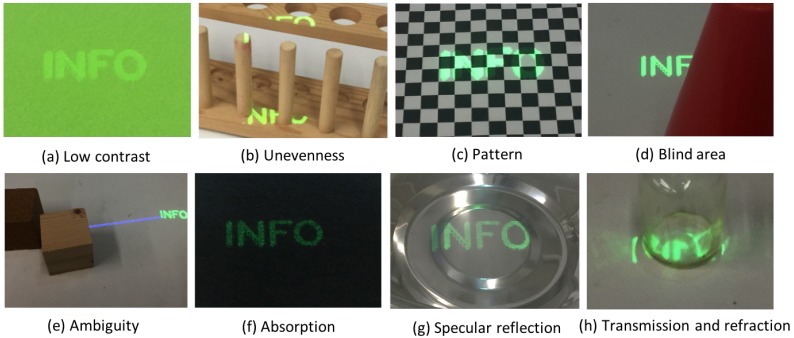
Categories of visibility degradation.

Ideal projection surface like a projection screen has white, flat, and high reflection surface. So, the projection surface that is far from such ideal condition may degrades the visibility. In this study, we consider eight factors of degradation as shown in Figure 3. Low contrast (a) means that the presented information is assimilated because the color difference or color brightness difference between the foreground information and background projection surface is small (a). Unevenness distorts information (b). A pattern in the projection surface (c) is caused by a large difference in the brightness of projection surface, which may divide the presented information. Occlusion (d) is caused by the positional relationship between the person and the object. An ambiguous annotation (e) confuses the viewer to identify the target of the label. This can also be considered as a result of blind area projection of a linkage line.

**Figure 4:**
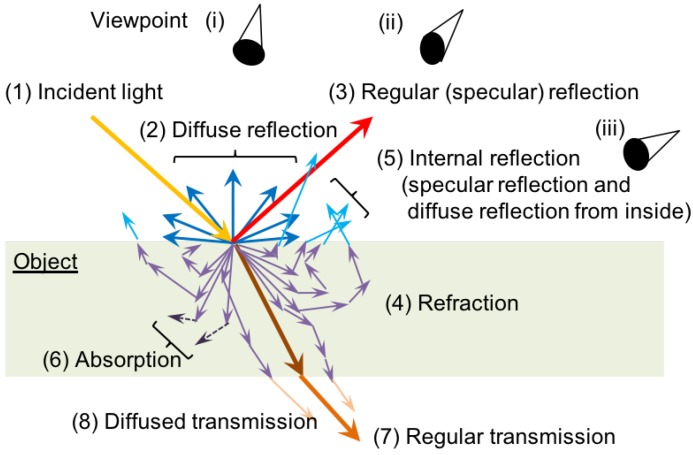
Basics of optics.

The cases of (f) absorption, (g) regular (specular) reflection, and (h) refraction and transmission represent the effects of the optical characteristics of the projection surface. Figure 4 illustrates these characteristics. In this figure, the incident light (1) is a light emitted from a projector onto the surface of an object, which causes (2) diffuse reflection, (3) regular (specular) reflection on the surface of the object. Also, the light is penetrated into the object with refraction (4), where the molecules of the object can cause complex refraction as well as regular reflection, and (5) internal reflection may appear on the surface again. Atoms in particular types of molecules absorb lights (6). The sum of these reflected lights is seen from a user or captured by a camera. So, in case of projection on the surface with high degree of absorption, the incident light is hardly seen. By contrast, when the information is projected on a surface with high degree of specular reflection such as metal and mirror, the regular reflection is dominant, and thus it is visible from the viewpoint located in the same direction as the reflection, e.g., Figure 4 (ii), but invisible from (i) and (iii), for example. The transmitted lights further appear as (7) regular transmission and (8) diffused transmission, which can be reflected on the other surface and visible as much more complex appearance as shown in Figure 3h.

### 3.4. Visibility Classes

**Figure 5:**
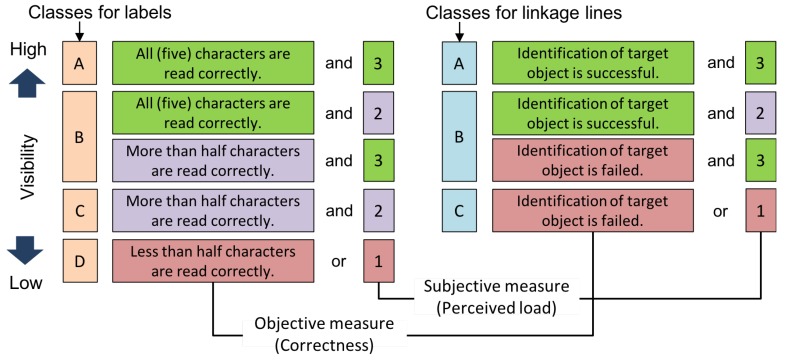
Definition of visibility classes for label and linkage line.

We represent “visibility” as a discrete class with an ordinal scale, which is assigned based on objective and subjective measures from human evaluators. The correctness of recognizing a text, i.e., recognition, and linking the label to an object are used as objective measures of visibility. By contrast, the subjective measure is provided by human evaluators how they feel the load of the presented tasks, which is chosen from 1 to 3 based on the criteria shown in Table 1. We used this subjective measure because we consider that the load of a task is difficult to measure by the correctness measure only. For example, the visibility is considered to be low if it takes too much time even though the information is correctly delivered to the person. Finally, four and three classes were specified for a label and a linkage line, respectively. The combination rule is presented in Figure 5. The visibility class estimator makes decision on the classes for a label and a linkage line separately, and the acceptability of a candidate position is judged based on the combination of the visibility estimations of a label and a linkage line as described in Section 3.1. For example, if the length of linkage line is 125 to 149 pixels and the estimated visibility classes for a label and a linkage line are “B” and “B”, respectively, the position is acceptable; however, in case that the length of a linkage line is less than 125 pixels, it is not acceptable.

## 4. Designing Features for Visibility Estimation

In this section, we design features for estimating visibility class.

### 4.1. Basic Flow of Feature Calculation

**Figure 6:**
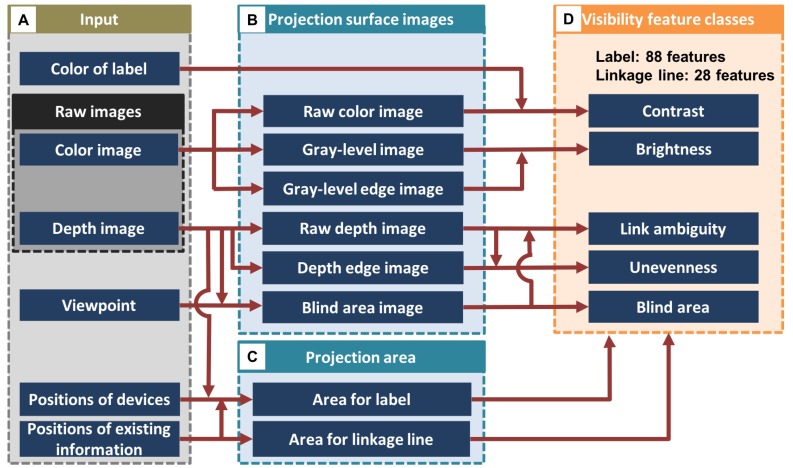
Processing flow in feature calculation.

Figure 6 summarizes the processing flow in feature calculation, which shows what kinds of information are used (A), what kinds of various types of intermediate information are calculated from input (B and C), and what kinds of visibility feature classes are obtained from the intermediate information (D).

#### 4.1.1. Input

The inputs to the feature calculation process are the RBG color of the characters in a label, raw images obtained from a color camera and a depth sensor, the viewpoint (the position of the center of the user’s eyes), the positions of devices (a color camera, a depth sensor, and a projector), and the positions of existing labels and linkage lines. The position is represented in a world coordinate system that takes the upper left corner as the origin O(0,0,0), in which the positions of the devices and the viewpoint are normalized by the resolution, i.e., pixel per inch (ppi), of a projector. These types of information are necessary to estimate visibility of projected information in a dynamic environment where the color and the position of projected information, the types and positions of objects on a table, the user’s position and height, and the configuration of devices are variable.

#### 4.1.2. Projection Surface Images

**Figure 7:**
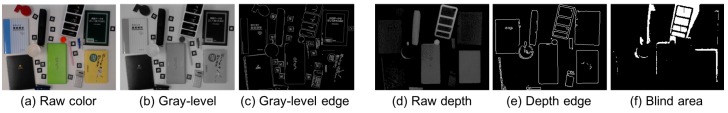
Intermediate images in feature calculation.

Two types of raw images, i.e., color and depth images, are converted into six types of intermediate images. Examples are shown in Figure 7. A raw color image (a) is a frame of images captured by a color camera, while an 8 bit gray-level image (b) is transformed from the raw color image. The Canny edge detector [37] is applied to a gray-level image to obtain an edge image (c). A depth image is used to represent the three dimensional characteristics of the projection surface, in which raw depth image (d) is merely a frame of depth images whose pixels represent the distance to corresponding points of objects from the depth camera. A depth edge image (e) is obtained by applying the Canny edge detector in the same manner as a gray-level edge image. A blind area image (f) is a binary image, in which black and white areas represent visible and invisible areas from a user’s viewpoint, respectively. The invisible area is computationally obtained by checking each pixel in the depth image if it can hide a particular point on the projection surface including other objects, which is based on the *planar projection shadow* method [38].

#### 4.1.3. Projection Area

**Figure 8:**
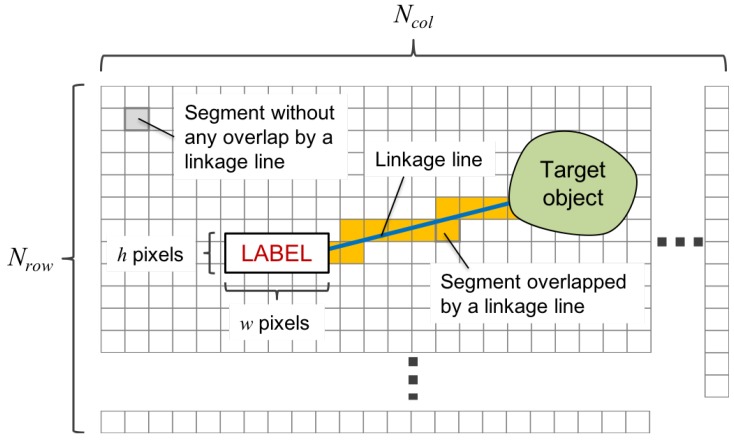
Definition of a region of interest (ROI) and a segment in a projection surface.

A projection area is defined by an area where the camera actually captures a label and a linkage line as rendered by the system. The projection area for a label is represented by a region of interest (ROI) of h×w pixels. By contrast, the projection area for a linkage line is represented as a collection of segments, in which a segment is a region when the projection image is divided into $N_{row}\times N_{col}$ regions. Figure 8 illustrates the definitions of ROI for a label and segments for a linkage line.

It should be noted that the position of a label captured by a camera could be different from what the system intended to present on the desk if the projection is overlapped on an object on the desk due to the height of the object. As described in Section 3.2, the relationship between the situation and the human’s evaluation is learned, in which “situation” is actually represented by a set of features obtained from a particular area in a camera image. Therefore, the area for calculating the features must reflect the area where the label is rendered in a camera image. Otherwise, the calculated features represent the situation that the label might not be included, and thus it may train the estimator using wrong relationship.

#### 4.1.4. Visibility Feature Classes

The features calculated from the projection surface images (Figure 6 B) within particular areas of a label and a linkage line (C) are categorized into five classes: contrast, brightness, link ambiguity, unevenness, and blind area. The contrast features represent the difference of colors between a label and the projection surface, as well as that of luminosity. The pattern and the reflection on the projection surface are characterized by the brightness features, which are obtained by the distribution of pixel brightness. The link ambiguity features indicate the degree of ambiguity in associating a label with a particular object based on the height of objects around the edge of the linkage line and the degree of occlusion by the objects. The unevenness features represent the shape of the projection surface, which are calculated in the same manner as brightness feature by regarding the depth image as a gray-level image. Finally, the blind area feature represents how much an ROI is occluded by an object. In total, 88 features and 28 features are defined for a label and a linkage line, respectively, which are presented in Section 4.2 and Section 4.3 in more detail.

### 4.2. Definition of the Features for a Label

In this section, the definitions of the features for a label are presented. Note that a complete list of the features with their informativeness are shown in Section 6.2. Some formulas are also used to calculate the features for a linkage line.

#### 4.2.1. Contrast Features

Color visibility is good if the color difference and color brightness difference between two colors are high, according to the Web Content Accessibility Guidelines 1.0 defined by W3C Web Accessibility Initiative (WAI) [39]. The color difference is defined by Formula (1), while Formula (2) represents the color brightness difference. Note that *R*, *B*, and *G* indicate the average color components in a ROI and that the suffixes *f* and *b* represent the foreground (label) and the background (projection surface), respectively.

(1) $$\begin{array}{ccc} CDIFF & = & |R_{f}-R_{b}\left|+\right|B_{f}-B_{b}\left|+\right|G_{f}-G_{b}| \end{array}$$

(2) $$\begin{array}{ccc} CBDIFF & = & 299\times |R_{f}-R_{b}\left|+587\times \right|B_{f}-B_{b}\left|+114\times \right|G_{f}-G_{b}| \end{array}$$

#### 4.2.2. Brightness Features

Two statistical features in a ROI of a gray-level image, i.e., average and variance, are defined by Formulas (3) and (4), respectively. Note that $p_{g}\left(i,j\right)$ represents the intensity of gray-level image at (*i*, *j*), and *N* is the number of pixels in a ROI, i.e., N=h×w. Also, the edge ratio (Formula (5)) represents the plausibility of edge of a ROI, where $N_{e}$ is the number of edge pixels in a gray-level edge image.

(3) $$\begin{array}{ccc} AVE_{g} & = & \frac{1}{N}\sum_{i=0}^{w-1}\sum_{j=0}^{h-1}p_{g}\left(i,j\right) \end{array}$$

(4) $$\begin{array}{ccc} VAR_{g} & = & \frac{1}{N}\sum_{i=0}^{w-1}\sum_{j=0}^{h-1}{\left(p_{g}\left(i,j\right)-AVE_{g}\right)}^{2} \end{array}$$

(5) $$\begin{array}{ccc} ER_{g} & = & \frac{N_{e}}{N} \end{array}$$

Shine and transparency of the projection surface is represented by three types of statistical values from the histogram of a gray-level image with $L_{gr}$ (=256) levels: variance, skewness, and kurtosis, which are defined by Formulas (6), (7), and (8), respectively [40]. In addition, these three types of features are calculated for a high frequency component image obtained by applying a 3 × 3 high-pass filter (Formula (9)). Note that, in these formulas, $\bar{\mu_{H}}$, $H_{i}$, and $\delta_{H}$ represent an average frequency in the histogram, the frequency in *i*-th bin (gray-level), and a standard deviation of frequency, respectively. Furthermore, the suffix *f* takes either high or all, indicating features from high frequency image and original image, respectively.

(6) $$\begin{array}{ccc} VAR_{H,f} & = & \frac{1}{N}\sum_{i=0}^{L_{gr}-1}{\left(H_{i}-\bar{\mu_{H}}\right)}^{2} \end{array}$$

(7) $$\begin{array}{ccc} SKEW_{H,f} & = & \frac{1}{N\times \delta_{H}^{3}}\sum_{i=0}^{L_{gr}-1}{\left(H_{i}-\bar{\mu_{H}}\right)}^{3} \end{array}$$

(8) $$\begin{array}{ccc} KURT_{H,f} & = & \frac{1}{N\times \delta_{H}^{4}}\sum_{i=0}^{L_{gr}-1}{\left(H_{i}-\bar{\mu_{H}}\right)}^{4} \end{array}$$

(9) $$\begin{array}{ccc} A & = & \left[\begin{array}{ccc} -1 & -1 & -1\\ -1 & 9 & -1\\ -1 & -1 & -1 \end{array}\right] \end{array}$$

Fractal geometry can be found in nature such as coastlines and mountains, which is characterized by “self-similarity”. A fractal dimension (FD) is utilized to quantify the degree of self-similarity. The larger the dimension becomes, the higher the self-similarity is. This indicates that the target is more complex, which has been applied in texture [41] and image analysis [42], as well as image segmentation [43] and recognition [44]. The box-counting dimension [45] is the most popular measurement of approximate fractal dimension due to its simplicity and computer-friendly nature. In the box-counting method, the number of cubes *r* pixel on a side, $N_{F}$, that cover the intensity surface of an ROI is counted, and a coefficient *D* in Formula (10) is estimated by the method of least squares against the double logarithm chart. In our system an ROI of 10 pixels square and scales *r* of 2, 3, 5, 10, and 18 pixels were utilized. Two types of FD are defined: $FD_{g}$ and $FD_{ge}$ for gray-level and gray-level edge images, respectively.

(10) $$\begin{array}{c} logN_{F}=-D_{k}logr+logC\;\;\left(k:g\;or\;ge,C:constant\right) \end{array}$$

To capture such a directional nature, we introduce features calculated from co-occurrence matrix and run-length matrix. Co-occurrence matrix proposed by Haralick et al. [46] is a matrix that represents the probability of existence of two points with certain intensity level at specific distance and angle. Let $p_{\theta }\left(i,j\right)$ be an element of a co-occurrence matrix for a direction θ (= 0, 45, 90, and 135 degrees) at (*i*, *j*). The distance between two points were set to 1, which means that only the neighboring pixels are taken into account for the calculation. In addition to the matrices for the four direction, an accumulated (and normalized) version of co-occurrence matrix is defined as the fifth one, i.e., θ = “sum”. Three types of co-occurrence matrix features proposed by Haralick et al. were used: sum of squares (SS), angular second moment (ASM), and inverse difference moment (IDM). In total, 15 features (=(4 directions + 1 “sum”)×3 types) were defined as co-occurrence matrix features. Sum of squares presents the smoothness of intensity surface consisting of neighboring pixels. As defined by Formula (11), the value gets larger as the number of pixel pairs with large difference of intensity level becomes larger. Angular second moment is defined by Formula (12), which represents the diversity of intensity level. The value increases as the number of pixel pairs with particular pixel difference gets large. Inverse difference moment represents the uniformity of the intensity distribution. As defined by Formula (13), the value gets larger as the difference between two points is small, i.e., looks uniform. Note that, prior to calculating the co-occurrence matrix, the level of a gray-level image is reduced by half of the original one ($L_{gr}$) to consider the processing speed ($L_{co}=L_{gr}/2=128$).

(11) $$\begin{array}{ccc} SS_{g,\theta } & = & \sum_{i=0}^{L_{co}-1}\sum_{j=0}^{L_{co}-1}{\left(i-j\right)}^{2}p_{\theta }\left(i,j\right) \end{array}$$

(12) $$\begin{array}{ccc} ASM_{g,\theta } & = & \sum_{i=0}^{L_{co}-1}\sum_{j=0}^{L_{co}-1}p_{\theta }{\left(i,j\right)}^{2} \end{array}$$

(13) $$\begin{array}{ccc} IDM_{g,\theta } & = & \sum_{i=0}^{L_{co}-1}\sum_{j=0}^{L_{co}-1}\frac{p_{\theta }\left(i,j\right)}{1+{\left(i-j\right)}^{2}} \end{array}$$

The other feature class regarding the continuity of pixel intensity is calculated from a run-length matrix. Run-length indicates the number of pixels with the same intensity level at a particular direction, which is originally utilized as an image coding method. A run-length matrix $r_{\theta }\left(i,j\right)$ is defined by the length of runs (*j*-th column) for an intensity level of *i* at direction θ (= 0, 45, 90, and 135 degree). Note that the column index of the matrix, i.e., *j*, starts with 1 according to the convention of the run-length matrix. We utilized five types of features proposed by Galloway [47]. In the following formulas, we decreased the intensity level of an image from $L_{gr}$ to $L_{rl}$ to avoid a sparse run-length matrix as well as to reduce the computational cost of features. $L_{rl}$ is specified by the larger edge of a ROI as represented in Formula (14). In addition, the maximum length of run is constrained by the larger edge of a ROI. So, the run-length matrix is represented as $L_{rl}\times L_{rl}$ matrix. $T_{g,\theta }$ (Formula (15)) represents the total number of runs for direction θ. Short runs emphasis (SRE) represents the amount of short runs (Formula (16)). The value decreases as short linear pattern appears. By contrast, long runs emphasis (LRE) represents the amount of long runs (Formula (17)). Gray level non-uniformity (GLN) represents unevenness of intensity level in a ROI (Formula (18)), while run-length non-uniformity (RLN) indicates the variance of the run length in a run-length matrix (Formula (19)). Finally, run percentage (RP) represents the ratio of the total number of runs to the number of pixels in an image (Formula (20)).

(14) $$\begin{array}{ccc} L_{rl} & = & max(h,w) \end{array}$$

(15) $$\begin{array}{ccc} T_{g,\theta } & = & \sum_{i=0}^{L_{rl}-1}\sum_{j=1}^{L_{rl}}r_{\theta }\left(i,j\right) \end{array}$$

(16) $$\begin{array}{ccc} SRE_{g,\theta } & = & \frac{1}{T_{g,\theta }}\sum_{i=0}^{L_{rl}-1}\sum_{j=1}^{L_{rl}}\frac{r_{\theta }\left(i,j\right)}{j^{2}} \end{array}$$

(17) $$\begin{array}{ccc} LRE_{g,\theta } & = & \frac{1}{T_{g,\theta }}\sum_{i=0}^{L_{rl}-1}\sum_{j=1}^{L_{rl}}j^{2}r_{\theta }\left(i,j\right) \end{array}$$

(18) $$\begin{array}{ccc} GLN_{g,\theta } & = & \frac{1}{T_{g,\theta }}\sum_{i=0}^{L_{rl}-1}{\left(\sum_{j=1}^{L_{rl}}r_{\theta }\left(i,j\right)\right)}^{2} \end{array}$$

(19) $$\begin{array}{ccc} RLN_{g,\theta } & = & \frac{1}{T_{g,\theta }}\sum_{j=1}^{L_{rl}}{\left(\sum_{i=0}^{L_{rl}-1}r_{\theta }\left(i,j\right)\right)}^{2} \end{array}$$

(20) $$\begin{array}{ccc} RP_{g,\theta } & = & \frac{T_{g,\theta }}{L_{rl}^{2}} \end{array}$$

In total, we obtain 46 features as brightness features: three statistical features from gray-level and gray-level edge images, three features from a gray-level histogram image, two fractal dimension features from gray-level and gray-level edge images, 15 features from co-occurrence matrices, and 20 features from run-length matrices.

#### 4.2.3. Unevenness Features

In the calculation of unevenness features, variance ($VAR_{d}$) and edge ratio ($ER_{d}$) are obtained by applying the depth image to Formulas (4) and (5), instead of gray-level image. Similarly, fractal dimensions for the depth-image and depth-edge image surfaces are calculated as $FD_{d}$ and $FD_{de}$, respectively. Furthermore, features derived from co-occurrence matrix and run-length matrix are calculated by Formulas (11) to (20), which are denoted $SS_{d,\theta }$, $ASM_{d,\theta }$, $IDM_{d,\theta }$, $T_{d,\theta }$, $SRE_{d,\theta }$, $LRE_{d,\theta }$, $GLN_{d,\theta }$, $RLN_{d,\theta }$, and $RP_{d,\theta }$. In total, 39 features are defined as unevenness features.

#### 4.2.4. Blind Area Feature

The blind area feature is defined as a ratio of the number of pixels in a blind area image ($N_{b}$) to the number of pixels in a ROI (Formula (21)).

(21) $$\begin{array}{ccc} BR & = & \frac{N_{b}}{N} \end{array}$$

### 4.3. Definition of Features for a Linkage Line

**Figure 9:**
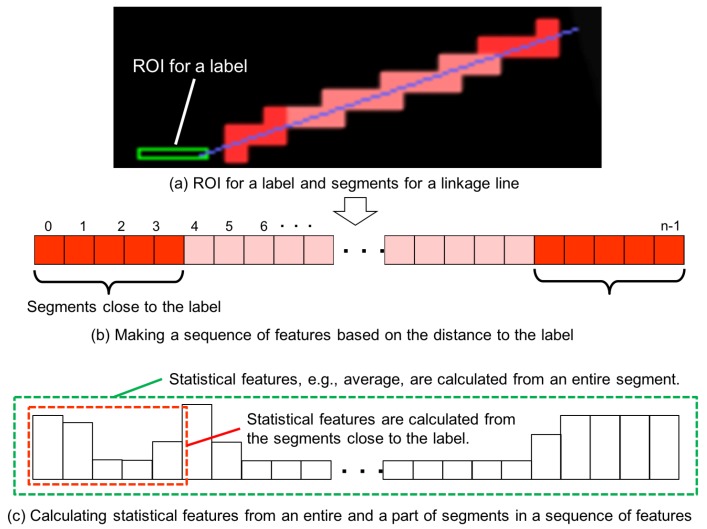
Definition of the features for a linkage line.

The features for the area of a linkage line are calculated by the following three steps, and Figure 9 illustrates the notion of the sequence and the sequence features.

**Step 1:**  Calculation of segment features
**Step 2:**  Making a sequence of segment features
**Step 3:**  Calculation of the linkage line features from the sequence data

In Step 1, 14 features are calculated for each segment, which is referred as segment features. A segment can be regarded as a ROI. So, the segment features are calculated in the same manner as the features for a label. Here, two color features (CDIFF and CBDIFF), nine brightness features ($AVE_{g}$, $VAR_{g}$, $ER_{g}$, $VAR_{H,all|high}$, $SKEW_{H,all|high}$, $KURT_{H,all|high}$), and the blind area feature (BR) represented by Formulas (1)–(8) and (21), are used as segment features from gray-level and gray-level edge images. In addition, two unevenness features from the depth-image are used: average ($AVE_{d}$) and edge ratio ($ER_{d}$) calculated by Formulas (3) and (5), respectively.

The next step (Step 2) is to make a sequence of segment features calculated in Step 1. A segment represents sorted *n*-segments based on the distance between the center of a label and that of a segment. Figure 9b shows an example of a sequence generated from the example of Figure 9a. As shown in (b), a sequence is represented by an array with *n*-elements, in which the first element is the closest segment to the label, while the last element is the one closest to the target object.

In Step 3, statistical features such as average, variance, skewness, and kurtosis are calculated for each sequence. For example, an average of the sequence data of CDIFF represents an average color difference in the segments of a linkage line. Furthermore, not only features for an entire sequence, but also for a particular portion close to the both ends are calculated because the features that relate to the visibility of the ends are important to avoid ambiguous linkage (Figure 3h). In general, the averages of segments features are used; however, higher order statistical features such as variance, skewness, and/or kurtosis are used to highlight the difference in the visibility resulting in the distribution of particular types of segment features, which includes the variance, skewness, and kurtosis of the average height ($AVE_{d}$) sequence and the variance of the average intensity of gray-level image ($AVE_{g}$). The naming convention of the linkage line features is represented below:

$${\left\{FSeg\right\}}_{portion,stat}$$

In the above, portion represents the portion of segment in a segment for calculation, which takes all, Lp, or Tp for the entire sequence, the *p*% segments closer to the label, and the *p*% segments closer to the target object, respectively. By contrast, stat takes ave, var, skew, or kurt for average, variance, skewness, and kurtosis, respectively. For example, the variance of the entire average height sequence is represented as ${\left\{AVE_{d}\right\}}_{all,var}$. A complete list of the features for a linkage line is presented in Section 6.2 with their informativeness.

## 5. Dataset for Training and Testing Visibility Estimator

In this section, we describe the data collection for building visibility estimator and data augmentation for balanced dataset.

### 5.1. Data Collection

**Figure 10:**
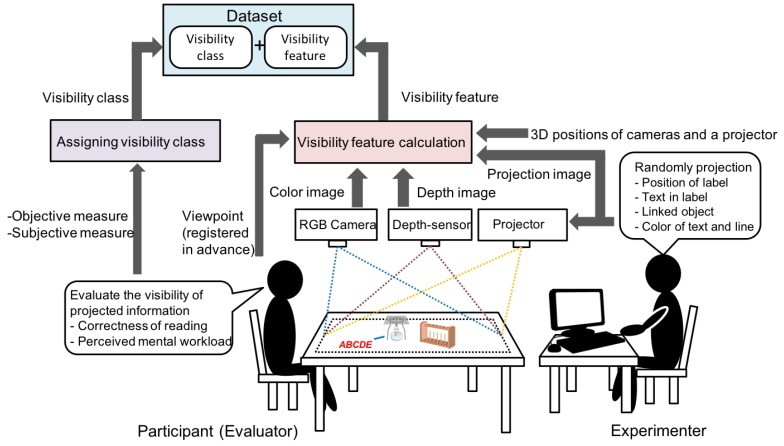
Data collection system.

**Figure 11:**
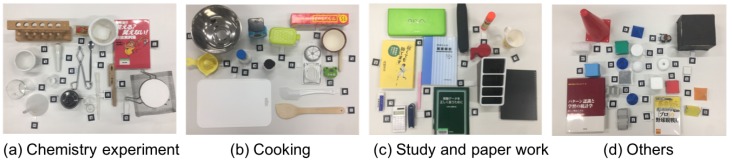
Four types of tabletop objects configurations.

Figure 10 illustrates how data collection is proceeded and how datasets for training and testing are generated. The data collection is carried out by a pair of persons: an evaluator and an experimenter, in which 15 visually healthy persons in their 20’s participated. Object configurations for four types of tabletop work were tested: a chemistry experiment, a cooking, a paperwork, and other tabletops (Figure 11).

A task of data collection is projecting information in a specific configuration of tabletop objects by the experimenter, followed by answering vocally by the evaluator what is presented and recording it by the experimenter. The projected information consists of a label with five capital alpha-numerical letters and its linkage line, in which the randomized elements are (1) the sequence of characters, (2) the position of the projection, (3) the linked object, and (4) the colors of the label and linkage line. Here, the colors are chosen from red, green, blue, yellow, and white. The presentation of information, i.e., the position of a label and the color of both the label and the linkage line is randomly selected every task. A set of tasks consist of 100 tasks, and the positions of objects on the projection surface are changed every set. For each tabletop work condition, an evaluator experienced two sets of tasks. Note that the experimenter puts the objects according to the system’s random choice of their positions for the first set in each task, while an evaluator arranged by him/herself in the second set.

The recorded information includes images from a color camera and a depth camera that consist of the projection surface. These images are stored for each task, which means that features of various projection conditions were calculated from eight images in total. Also, the three dimensional position of the estimator’s head is measured in advance so that it could be represent their viewpoints. In total, 800 pairs of estimation features of projection surface and their corresponding visibility are collected for each evaluator, and thus 12,000 pairs from 120 tabletop objects configuration are used.

Throughout a task, both objective and subjective measures of visibility are gathered as described in Section 3.4. The correctness of recognizing a text and linking the label to an object are used as objective measures of visibility. Note that fiducial makers with numbers are used not only to identify the position of objects, but also to let the evaluators tell the printed number as what they consider linked to a particular label. By contrast, the subjective measure is provided by the evaluators how they feels the load of the presented tasks based on the criteria shown in Table 1.

Hardware configuration is as follows: a Logicool HD Pro Webcam C920 is used as an RGB camera, while Microsoft Kinect v2 is utilized as a depth-sensor. A video projector is EPSON EB1725. A Windows 10 PC (CPU: Intel Core i7-6700, Memory: 8 GB) runs data collection system. The dimension of the projection surface is 71 cm × 51 cm. The net RGB camera resolution is 960 × 720 pixels (34.3 ppi) and the net depth-camera resolution is 286 × 216 pixels (10.2 ppi). The system is also used for online user experiment described in Section 6.3.

### 5.2. Data Augmentation for Balanced Dataset

We found that the number of instances calculated from ROIs within blind areas and on uneven areas is small based on analysis of the distribution of the blind area ratio (BR), the variance of depth values ($VAR_{d}$), and the depth edge ratio ($ER_{d}$). Therefore, we synthesized ROIs based on an original ROI with the values of more than 0.04, 0.24, and 0.04 of BR, $VAR_{d}$, and $ER_{d}$, respectively, in which an original ROI was slid into a random direction by 5% of its width or 100% of its height.

As described above, 12,000 pairs of projection patterns and associated evaluations by human evaluators were collected; however, the number of instances in each class is imbalanced as shown in Table 2, in which 3.1 times and 16.9 times between the largest and smallest ones in the label and the linkage line, respectively. The instances in each class of the label were either over-sampled or under-sampled so that the numbers could be 2500, while the number for the linkage line is set to be 600. We utilized Syntactic Minority Over-sampling Technique (SMOTE) and SpreadSubsample filters in WEKA machine learning toolkit [48], respectively.

## 6. Evaluation

In this section, the visibility estimator is evaluated.

### 6.1. Difference in Various Models of Classifiers

An offline experiment is conducted to understand the basic classification performance of the visibility classifier.

#### 6.1.1. Methodology

Popular classifier models were compared in both label and linkage line visibility estimation, which includes RandomForests (RF), Support Vector Machines (SVM), Nearest Neighbor (NN), and Naïve Bayes (NB). The WEKA machine learning toolkit was used in this experiment. The number of trees in RF for classifiers of both label and linkage line was set to 100 by taking into account the classification performance and processing speed. In training SVM, we used Sequential Minimal Optimization (SMO) with the major hyper-parameters of Gaussian Radial Basis Function Network (RBFNetwork) as a kernel function and 1.0 as a gamma value. Regarding the complexity parameters (*C*), 100.0 and 10.0 were used for a label visibility estimator and a linkage line estimator, respectively. Both the gamma valuee and the complexy parameters were chosen using a grid search. We performed 10 fold cross-validation to see average performance of the classifiers.

#### 6.1.2. Result

Table 3 summarizes the F-measures of various classifier models for label visibility classification. F-measure (22) is a harmonic mean between recall (23) and precision (24), where the suffix *i* indicates the visibility classes (i∈{A,B,C,(D)}), and $N_{correct}$, $N_{tested}$, and $N_{judged}$ represent the number of instances correctly classified as class *i*, the total number of instances in class *i*, and the number of instances judged as class *i*, respectively. The F-measures in Table 3 are the average F-measures over all classes. As shown in the table, RandomForest (RF) is the best classification model in the four models, and its breakdown is shown as a confusion matrix in Table 4.

(22) $$\begin{array}{ccc} F_{i} & = & \frac{2}{1/recall_{i}+1/precision_{i}} \end{array}$$

(23) $$\begin{array}{ccc} recall_{i} & = & \frac{N_{correct_{i}}}{N_{tested_{i}}} \end{array}$$

(24) $$\begin{array}{ccc} precision_{i} & = & \frac{N_{correct_{i}}}{N_{judged_{i}}} \end{array}$$

The performance of linkage line classification per classes are carried out on four levels of segment resolution (Table 5). As described in Section 4.3, a segment is one of areas obtained by diving the projection surface into $N_{row}\times N_{col}$ areas. When the number of segments in a projection surface increases, each segment represents more local features. The table shows that RandomForest performed best in the four models against all levels of segment resolution; especially, RandomForest with features calculated from 36 × 48 segments was the best (F-measure is 0.789). Therefore, in the later experiments, we use this configuration. The breakdown is shown in Table 6.

### 6.2. Feature Subset Evaluation

In Section 4, 88 and 28 feature were specified as those characterize the visibility of a label and a linkage line, respectively. To improve the online processing speed and avoid over-fitting of the trained classifiers, feature selection (attribute selection) was conducted.

#### 6.2.1. Methodology

We took a wrapper approach, in which a particular classifier, i.e., RandomForest with 100 trees, was used to evaluate the effectiveness of a subset of features based on its classification accuracy. Greedy forward search method was used to find the best subset of features by adding the most effective feature one-by-one. The subset that does not increase the accuracy anymore is finally regarded as the best one. In addition to identifying feature subsets, we calculated information gain (IG) as an indicator of informativeness of each feature, where the gain of information provided by a particular feature is calculated by subtracting a conditional entropy with that feature from the entropy under random guess [49]. So, the more informative feature has the higher IG. Furthermore, the elapsed time for a series of view management was measured, in which the elapsed time of feature calculation was compared before and after the feature selection.

#### 6.2.2. Results and Discussion

A total of 15 features was selected as the best subset for classifying label visibility with RandomForest classifier. Table 7 shows a complete list of features for a label with the type, indication of selected feature (a check mark ✓ means that it was selected), and IG. The table implies that the degree of blind area (BR) of a certain ROI is the most informative in the classification. The table also shows that IDM tends to be informative both in the gray-level image and the depth-image. As described in Section 4.2.2 and Section 4.2.3, $IDM_{g}$ and $IDM_{d}$ represent the uniformity of gray-scale appearance and the unevenness of the surface in a ROI, respectively. Other gray-level non-uniformity feature ($GLN_{g}$) and unevenness feature ($ASM_{d}$) that represents the diversity of the projection surface were also informative. The informativeness of these features implies that not only visually uniform but also physically even is important for effective (visible) presentation.

Regarding the linkage line, 14 features were selected as the best subset, and Table 8 shows a complete list of the linkage line features with an indicator of selection. In the table, $BR_{T}$ features are three most informative features, which indicates the visibility of the linkage line on the target object side is important. The features $\left\{AVE_{d}\right\}$ also have high information gain, which we consider a particular value indicating zero-height, i.e., on the table, tends to be high visibility. Although the IG of CBDIFF is 0.000 bit, it was chosen as an element of best subset. To examine its value, we removed it from the feature subset. The resultant F-measure was 0.728, which was smaller than the original subset by 0.033. So, we concluded that it contributes to classification performance as a whole although it is not effective as a single feature.

In both a label and a linkage line, the histogram features $VAR_{H}$, $SKEW_{H}$, and $KURT_{H}$ were generally little informative, especially, high frequency range with a suffix of high, as well as contrast features CDIFF and CBDIFF. The histogram features for all frequency components, i.e., original image, are intended to represent the shine of the projection surface, while the high frequency components are for transparency. The contrast features were designed to represent the contrast between the projection surface and the projected information. We consider that the low informativeness of these features does not directly indicate that these features are useless. As described in Section 6.3, the cases where the system over-estimated the label visibility class are low contrast and projected information transmitted through transparent material. So, the formulas for these features have rooms for improvement.

Table 9 and Table 10 show the confusion matrices for a label and a linkage line visibility classifiers in the configurations of selected features, respectively. From these tables, we can confirm that the classification performance of a label was decreased from 0.919 to 0.913 and that of a linkage line was decreased from 0.789 to 0.761. Although selected features decreased classification performance, i.e., F-measure, we consider that the negative impact on the user’s task performance is limited, rather the decreased number of features contributes to processing speed.

Table 11 summarizes the processing speed of calculating features before and after the feature selection, which suggests that the processing speed was improved by 173% (=78.8/45.5 × 100) through the feature selection. Note that the area of ROI depends on the size of information to be presented, and that the number of segments to be considered in calculating the features for a linkage line also varies by the length of the line. In VisLP algorithm, candidates of label placement are tested around a target object every 10 degrees, and the linkage line is extended if no suitable label position is found. The classification by RandomForest classifier took 0.003 ms. So, every check of candidate positions takes about 46 ms. Another time consuming processing in a view management is making projection surface images (Figure 6B), which took 100 ms in total; however, this calculation is performed only once for each opportunity of view management. Therefore, the bottleneck of the system is feature calculation, and the feature selection contributed in reducing the entire processing time. However, the processing time for feature calculation should be reduced for real-time label placement.

### 6.3. Online Experiment with Users

In this section, an online evaluation of visibility classifiers is carried out, which works as a “test” phase in the machine learning context.

#### 6.3.1. Methodology

**Figure 12:**
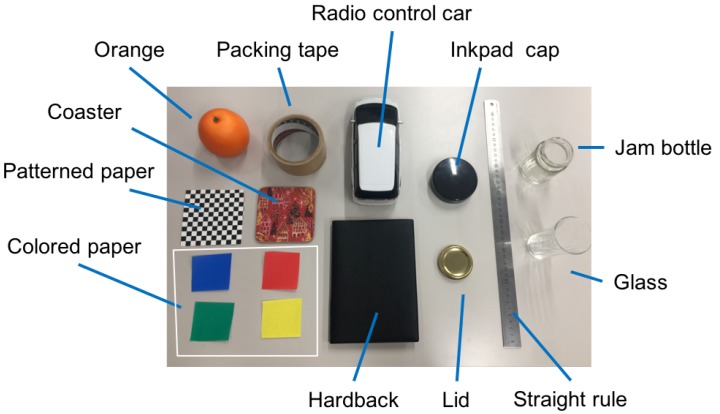
Physical objects used in the online experiment.

The experiment was carried out basically in the same manner as data collection described in Section 5.1 except for the physical objects on the desk and the participants. We used 15 objects that were not used in the data collection (Figure 12), and 10 visually healthy persons in their 20’s who did not participate in the data collection were recruited. Therefore, the trained classifiers knew neither the objects nor the participants, which allows us to understand a practical performance.

A task consists of a pair of presentations of information and evaluation of visibility based on the criteria shown in Table 1. Over-estimation occurs when the level of visibility obtained from the participant is lower than the one estimated by the system. In such a case, the participant was asked the reason for his/her judgment. Each participant performed 10 tasks. At each task, the experimenter randomly changed the presentation of information; however, the layout of the physical objects were randomly changed once per participant. Note that the visibility classifiers for a label and a linkage line were trained with the dataset collected in Section 5.1 for an implementation of RandomForests (RandomTree) in the OpenCV 3.0 (C++) platform. In addition, the participant’s viewpoint, i.e., 3D coordinates in the system coordinates, was registered with the system in advance.

#### 6.3.2. Results and Discussion

The confusion matrices of the visibility classification of a label and a linkage line are shown in Table 12 and Table 13, respectively. Note that the number of instances in each class is not normalized to understand an actual classification performance. So, the performance metrics, i.e., recall, precision, and F-measure, cannot be compared with the ones presented in Section 6.1 and Section 6.2.

Formula (25) defines “estimation gap” as the difference between the participant’s evaluation ($V_{p}$) and the system’s estimation ($V_{s}$). Here, we assume that the visibility classes (Figure 5) represent the level of visibility at a regular interval with values of 4, 3, 2, and 1 for classes “A”, “B”, “C”, and “D”, respectively. Therefore, the gap with a negative value such that $V_{p}$ is “B” and $V_{s}$ is “A” is regarded as over-estimation with a gap value of “−1”. By contrast, under-estimation is a situation where the gap has a positive value. The zero gap is an ideal case in which the participant’s evaluation and the system’s estimation are identical.

(25) $$\begin{array}{ccc} Gap & = & V_{p}-V_{s}\;\;\;,where\;V_{p},V_{s}\in \left\{A,B,C,D\right\} \end{array}$$

**Figure 13:**
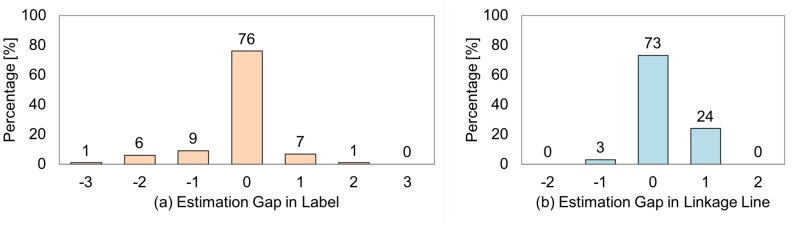
The distribution of gaps between the participant’s evaluation and the system’s estimation.

**Figure 14:**
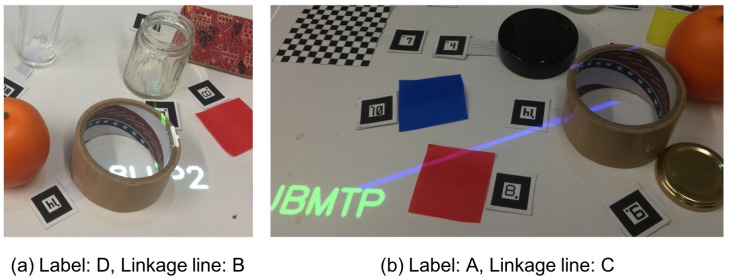
Examples of resultant projection that matches the participants’ evaluations with the system’s decisions, e.g., zero-gaps. The pictures were taken from the participants’ viewpoints.

Figure 13 shows the relative frequency distributions for the label and the linkage line presentation calculated from Table 12 and Table 13 using Formula (25). As shown in the figure, 76% of the presentations of labels and 73% of linkage line presentations were matched with the participants’ evaluations. Figure 14 shows examples of situations where the participants’ evaluations and the system’s judgments were identical, i.e., zero-gaps. In (a), the projected text is deformed and partially hidden due to an overlap with the packing tape, which we consider was successfully represented in the features and judged as “D”. One end of the linkage line is also overlapped with the packing tape; however, it was not so large that degraded the entire visibility. So, we consider that it was judged as “B”. In (b), the label is clearly seen, and thus it should be judged as “A”; however, the linkage line is ambiguous because both a packing tape and an orange are on the same line, which we consider that it should be judged as “C”. Therefore, the features that represent a situation where an end of a linkage line is hidden in a blind area worked effectively.

By contrast, 24% of label placement and 27% of linkage line placement were classified incorrectly. In terms of a label, over-estimation is more likely to happen than under-estimation. Over-estimation is a situation where the participants’ evaluation is worse than the system’s judgment. In other words, the participant did not feel so comfortable as the system expected. Thus, it is more critical than the case of under-estimation. Table 14 summarizes the reasons for lower evaluations of the participants than the system’s estimations. Regarding the label, the most frequent reasons for the gap value of “−1” were the deformation of presented label due to the unevenness of the projection surface, followed by difficulty in reading caused by low contrast.

**Figure 15:**
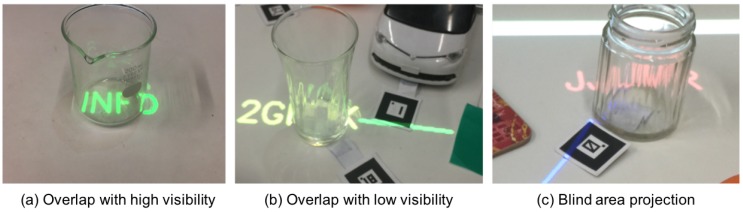
Examples of projection on and behind glass objects.

Transmission and refraction is the most common reason in all gaps. The situation happened when projected light overlapped with glass objects or the label was projected behind glass objects. As described in Section 3.3, incident light can be transmitted through an object and seen through from a user, in which a glass is a typical case. However, refraction inside the glass as well as at the boundary between the air and the glass can generate complex light paths. Figure 15 shows examples of projection on and behind glass objects. The projected information can be clearly seen in one case (a), but, in the other case (b), it is hard to see through. Also in (c), the label is projected behind a glass object, i.e., in the blind area, and its visibility is affected in the same manner. We consider that the reason for failing in estimating such situations as low visibility (“C” or “D”) comes from the characteristics of depth-sensing. We used Microsoft Kinect V2 as a depth sensor, which employs the Time-of-Flight (ToF) method for depth-sensing and requires proper reflection of Infra-red light. However, the emitted infra-red light is also affected by transmission and refraction. So, the depth information of glass objects cannot be handled correctly. In addition to the technical issue, we did not use such glasses with deformed and somehow translucent surface as shown in Figure 15b,c when we collect data for training classifiers, rather a beaker and conical flask as shown in Figure 11a were used. Therefore, the classifiers were not trained with data collected under such a tough environment. Actually, two of four cases of “being hidden in the blind area” as a reason in the gap value of “−2” were caused by the situation of Figure 15c.

As for the linkage line, under-estimation is a major erroneous classification, which means that the association was more correct and easy for participants than the system expected. This is not so bad as the case of over-estimation. There were three cases of over-estimation. The reasons provided by the participants was shown in Table 14, where ambiguous annotation target, difficulty in associating the label with the target due to unevenness of the projection surface, and transmission and refraction of the projected line were the reasons for lower evaluations than the system’s judgments.

## 7. Conclusions

In this article, we proposed a view management method for spatial augmented reality based on machine-learning. The motivation of the work is to find the position of an annotation (label and associated linkage line) while taking into account its visibility affected by the reflective characteristics of the projection surface, the three dimensional properties of physical objects on the projection surface, and the spatial relationship between the objects, the projector-camera systems, and the user. A weighted linear combination of factors that affect visibility is often utilized in view management; however, the methods of finding optimum weights have not been shown or dependent on the application designers. Also, existing approaches try to find suitable positions for labels and linkage lines, in which “suitability” is not truly defined from the user’s point of view, i.e., it is not clear how the user feels the situation and what is expected in the user’s behavior. To address the issues, a supervised machine-learning technique was applied to model the visibility of information with human’s subjective and objective measures.

We defines the machine-learning task as a classification problem with four classes for a label visibility estimation and three classes for a linkage line. We collected data from 15 visually healthy persons, which consists of 12,000 instances from 120 tabletop object configurations in four different work situations. For the two classifiers, we defined 88 and 28 features, respectively, and feature selection specified 15 and 14 best feature subset. The F-measures for the classification of a label and a linkage line are 0.913 and 0.761, respectively. We confirmed the processing speed improvement of 173% with degradation of classification performance of 0.7% and 3.5%, respectively. Considering the benefit of speed improvement, the performance degradation is acceptable. The judgment of one candidate position takes about 46 ms, which may become an issue if the system is in a cluttered dynamic environment and is required a quick response to the change. By contrast, if a good condition where no tall objects exists and reflective characteristics of the surface are ideal, the current processing speed may be enough.

In an online-evaluation, we tested against 10 new persons with 15 new objects to show the robustness of the classifiers against unknown users and objects. The results showed that 76.0% of the system’s judgments were matched with the participants’ evaluation for a label visibility estimation, while that of a linkage line was 73.0%. Over-estimation, in which the system’s judgments were better than human evaluations and more critical than under-estimation, was observed in 16.0% of label visibility estimations.

One of future work is the improvement of over-estimation. We need to investigate a method that measures the shape of glass objects precisely, as well as collecting data from objects with more heterogeneous appearances and materials. However, to obtain large amount of data from human participants, we need to consider the efficiency. In the presented work, each participant comes to the lab, reads the presented text, and rates its subjective visibility of a label and a linkage line 800 times, which is very burdensome. We consider the number of participants can be increased and the time to take the data collection can be shortened if an online survey method is investigated for crowd-sourcing. To realize this, visibility estimation that does not depend on the resolution and the display size of online participants terminals, which is another future work. Furthermore, the viewpoint estimation should be automated, which is currently registered manually, and the participants in the data collection were asked to be stable during the experiment. To address this issue, an automatic gaze estimation functionality should be incorporated into the system.

## Figures and Tables

**Table 1:** Criteria for subjective measures.

Score	Criteria for a Label	Criteria for a Linkage Line
1	I cannot understand more than half of the characters.	I cannot identify the target object.
2	I can understand a couple of characters,	I am not confident of the target object
	or it takes some time to understand all characters.	although I think I can identify it,
		or it takes some time to identify the target.
3	I can understand all characters immediately.	I can identify the target object immediately.

**Table 2:** The numbers of instances in each class.

Type	Label				Linkage Line		
Class	A	B	C	D	A	B	C
Number of samples (Original)	5712	2349	1868	2071	8816	2663	521
Number of samples (Balanced)	2500	2500	2500	2500	600	600	600

**Table 3:** F-measures for label visibility classification.

RF	SVM	NN	NB
0.919	0.912	0.911	0.530

**Table 4:** Confusion matrix of label visibility classification with RandomForest (RF).

		Classified as				Recall
		A	B	C	D	
Original	A	2360	19	116	5	0.944
	B	46	2258	95	101	0.903
	C	133	134	2211	22	0.884
	D	20	99	22	2359	0.944
	Precision	0.922	0.900	0.905	0.949	F: 0.919

**Table 5:** F-measures for linkage line visibility classification.

Segment Resolution ($N_{\mathrm{row}}\times N_{\mathrm{col}}$)	RF	SVM	NN	NB
72 × 96	0.782	0.742	0.705	0.539
36 × 48	0.789	0.732	0.721	0.559
24 × 32	0.774	0.702	0.700	0.547
18 × 24	0.745	0.721	0.708	0.553

**Table 6:** Confusion matrix of linkage line visibility classification with RF.

		Classified as			Recall
		A	B	C	
Original	A	485	57	58	0.808
	B	76	453	71	0.755
	C	58	60	482	0.803
	Precision	0.784	0.795	0.789	F: 0.789

**Table 7:** List of the features for a Label (a check mark ✓ indicates that the feature was selected).

Name	Type	Sel.	IG [bit]	Name	Type	Sel.	IG [bit]	Name	Type	Sel.	IG [bit]
BR	BA	✓	0.737	$LRE_{d,45}$	UE		0.312	$VAR_{g}$	BR		0.184
$IDM_{d,all}$	UE		0.469	$LRE_{d,0}$	UE		0.307	$ASM_{g,0}$	BR		0.184
$IDM_{d,135}$	UE		0.451	$RLN_{d,90}$	UE		0.304	$ASM_{g,135}$	BR		0.183
$IDM_{g,all}$	BR	✓	0.448	$LRE_{d,90}$	UE		0.302	$ASM_{g,45}$	BR		0.176
$IDM_{g,45}$	BR		0.447	$RP_{d,135}$	UE		0.300	$ER_{g}$	BR	✓	0.164
$IDM_{g,135}$	BR		0.446	$GLN_{d,45}$	UE		0.298	$SRE_{g,0}$	BR		0.147
$IDM_{g,90}$	BR	✓	0.439	$RP_{g,0}$	BR		0.285	$LRE_{g,90}$	BR		0.142
$IDM_{d,45}$	UE	✓	0.438	$GLN_{g,0}$	BR		0.283	$LRE_{g,0}$	BR		0.141
$IDM_{d,0}$	UE	✓	0.438	$RLN_{g,0}$	BR		0.278	$LRE_{g,45}$	BR		0.140
$IDM_{g,0}$	BR	✓	0.435	$SRE_{d,90}$	UE		0.277	$SRE_{g,90}$	BR		0.139
$ASM_{g,all}$	BR		0.435	$SRE_{d,0}$	UE		0.273	$LRE_{g,135}$	BR		0.136
$ER_{g}$	UE		0.410	$RP_{d,45}$	UE		0.269	$AVE_{g}$	BR		0.135
$IDM_{d,90}$	UE	✓	0.408	$RLN_{d,135}$	UE		0.264	$SRE_{g,135}$	BR	✓	0.127
$VAR_{d}$	UE		0.402	$RLN_{g,90}$	BR		0.259	$SRE_{g,45}$	BR		0.125
$ASM_{d,135}$	UE		0.392	$SRE_{d,45}$	UE		0.255	$SKEW_{H,all}$	BR		0.123
$GLN_{g,135}$	BR		0.392	$RLN_{d,45}$	UE		0.253	$KURT_{H,all}$	BR		0.107
$GLN_{g,45}$	BR		0.390	$FD_{d}$	UE		0.248	$KURT_{H,high}$	BR		0.105
$ASM_{d,45}$	UE		0.378	$SRE_{d,135}$	UE		0.247	$SKEW_{H,high}$	BR		0.103
$ASM_{d,0}$	UE		0.376	$RLN_{g,135}$	BR	✓	0.239	$FD_{e}$	BR		0.079
$RLN_{d,90}$	UE		0.375	$RP_{d,90}$	UE	✓	0.237	$SS_{g,135}$	BR	✓	0.053
$GLN_{g,90}$	BR		0.371	$RLN_{g,45}$	BR		0.233	$SS_{g,all}$	BR		0.052
$ASM_{d,90}$	UE		0.360	$SRE_{d,90}$	UE	✓	0.233	$SS_{g,0}$	BR		0.052
$RP_{d,0}$	UE		0.359	$SS_{d,135}$	UE		0.221	$SS_{g,45}$	BR		0.049
$FD_{de}$	UE	✓	0.347	$SS_{d,45}$	UE		0.219	$SS_{g,90}$	BR		0.048
$FD_{ge}$	BR	✓	0.344	$SS_{d,0}$	UE		0.216	$VAR_{H,all}$	BR		0.047
$LRE_{d,0}$	UE		0.342	$SS_{d,all}$	UE		0.216	$VAR_{H,high}$	BR		0.046
$RP_{g,45}$	BR		0.333	$SS_{d,90}$	UE		0.209	CBDIFF	CN		0.013
$GLN_{d,135}$	UE		0.331	$RP_{g,90}$	BR		0.197	CDIFF	CN		0.009
$RP_{g,135}$	BR		0.326	$ASM_{d,90}$	UE		0.187				
$LRE_{d,135}$	UE		0.317	$ASM_{d,all}$	UE		0.184				

**Table 8:** List of the features for a linkage line (a check mark ✓ indicates that the feature was selected).

Name	Type	Sel.	IG [bit]	Name	Type	Sel.	IG [bit]
${\left\{BR\right\}}_{T10,ave}$	BA	✓	0.224	${\left\{AVE_{d}\right\}}_{all,kurt}$	UE		0.074
${\left\{BR\right\}}_{T20,ave}$	BA	✓	0.220	${\left\{BR\right\}}_{L10,ave}$	BA	✓	0.068
${\left\{BR\right\}}_{T30,ave}$	BA	✓	0.205	${\left\{VAR_{H,high}\right\}}_{all,ave}$	BR		0.067
${\left\{BR\right\}}_{all,ave}$	BA	✓	0.136	${\left\{BR\right\}}_{L30,ave}$	BA		0.060
${\left\{AVE_{d}\right\}}_{T10,ave}$	UE	✓	0.127	${\left\{SKEW_{H,high}\right\}}_{all,ave}$	BR		0.057
${\left\{ER_{d}\right\}}_{all,ave}$	UE		0.125	${\left\{AVE_{d}\right\}}_{L20,ave}$	UE	✓	0.050
${\left\{AVE_{d}\right\}}_{T30,ave}$	UE	✓	0.124	${\left\{KURT_{H,high}\right\}}_{all,ave}$	BR		0.047
${\left\{AVE_{d}\right\}}_{T20,ave}$	UE		0.120	${\left\{AVE_{g}\right\}}_{all,ave}$	BR	✓	0.043
${\left\{AVE_{d}\right\}}_{all,var}$	UE	✓	0.096	${\left\{AVE_{d}\right\}}_{L10,ave}$	UE	✓	0.040
${\left\{VAR_{H,all}\right\}}_{all,ave}$	BR		0.091	${\left\{ER_{g}\right\}}_{all,ave}$	BR		0.037
${\left\{SKEW_{H,all}\right\}}_{all,ave}$	BR		0.083	${\left\{AVE_{d}\right\}}_{L30,ave}$	UE		0.036
${\left\{KURT_{H,all}\right\}}_{all,ave}$	BR	✓	0.082	${\left\{AVE_{g}\right\}}_{all,var}$	BR	✓	0.014
${\left\{AVE_{d}\right\}}_{all,skew}$	UE		0.081	${\left\{CBDIFF\right\}}_{all,ave}$	CN	✓	0.000
${\left\{BR\right\}}_{L20,ave}$	BA		0.077	${\left\{CDIFF\right\}}_{all,ave}$	CN		0.000

**Table 9:** Confusion matrix of label visibility classification with selected 15 features.

		Classified as				Recall
		A	B	C	D	
Original	A	2353	22	121	4	0.941
	B	23	2267	119	91	0.907
	C	130	169	2183	18	0.873
	D	14	124	32	2330	0.932
	Precision	0.934	0.878	0.889	0.954	F-measure: 0.913

**Table 10:** Confusion Matrix of Linkage Line Visibility Classification with Selected 14 Features.

		Classified as			Recall
		A	B	C	
Original	A	461	78	61	0.768
	B	84	457	59	0.762
	C	76	72	452	0.771
	Precision	0.742	0.753	0.790	F: 0.761

**Table 11:** Comparison of processing speed of feature calculation before and after feature selection (assuming that the physical area of a region of interest (ROI) and the length of the linkage line are 10 cm × 2 cm and 10 cm, respectively).

	Label (ms)	Linkage Line (ms)	Total (ms)
Before Selection	78	0.8	78.8
After Selection	45	0.5	45.5

**Table 12:** Confusion matrix of label visibility classification in offline experiment.

		Classified as				Recall
		A	B	C	D	
Original	A	36	6	0	0	0.864
	B	8	14	0	1	0.609
	C	3	1	1	1	0.167
	D	1	3	0	23	0.852
	Precision	0.760	0.583	1.000	0.920	F: 0.643

**Table 13:** Confusion matrix of linkage line visibility classification in online experiment.

		Classified as			Recall
		A	B	C	
Original	A	50	22	0	0.694
	B	2	21	2	0.840
	C	0	2	2	0.667
	Precision	0.961	0.477	0.500	F: 0.662

**Table 14:** The reasons provided by the participants when the system over-estimated. The numbers in the brackets indicate the number of answers.

Gap	Label	Linkage line
−1	Unevenness (4), Low contrast (2),	Ambiguous annotation target (1), Unevenness (1)
	Transmission and refraction (2),	Transmission and refraction (1)
	Absorption and pattern (1)	
−2	Blind area (4), Unevenness (1),	
	Transmission and refraction (1)	
−3	Transmission and refraction (1)	–
